# External validation of the BABY‐Knee Algorithm for treatment decision‐making in skeletally immature patients with anterior cruciate ligament injuries: A Dutch cohort study

**DOI:** 10.1002/jeo2.70891

**Published:** 2026-08-18

**Authors:** Annika Babette Ito, Rob Paulus Augustinus Janssen, Stijn Thomas Henricus Croes, Robin Nicolaas Julian Voskuilen, Alberto Grassi, Maria Christine van der Steen, Martijn Dietvorst

**Affiliations:** ^1^ Department of Orthopaedic Surgery and Trauma Máxima Medical Centre Veldhoven the Netherlands; ^2^ Department of Biomedical Engineering Eindhoven University of Technology, Orthopaedic Biomechanics Eindhoven the Netherlands; ^3^ Department of Paramedical Sciences Fontys University of Applied Science Eindhoven the Netherlands; ^4^ Dipartimento di Scienze Biomediche e Neuromotorie (DIBINEM) Università di Bologna Bologna Italy; ^5^ Clinica Ortopedica e Traumatologica II, Istituto Ortopedico Rizzoli Bologna Italy; ^6^ Department of Orthopaedic Surgery and Trauma Catharina Hospital Eindhoven the Netherlands

**Keywords:** 2018 IOC consensus statement, ACL injury, BABY‐Knee Algorithm, decision support, paediatric

## Abstract

**Purpose:**

The aim of this study was to investigate the external validity of the Best anterior cruciate ligament (ACL)‐treatment Based on the Years of the Knee (BABY‐Knee) Algorithm, developed based on an Italian population and to assess its applicability to a Dutch patient population treated in accordance with the 2018 International Olympic Committee (IOC) consensus statement.

**Methods:**

A total of 109 skeletally immature patients with ACL injuries were included from the Dutch contribution to the European Society of Sport Traumatology, Knee Surgery and Arthroscopy (ESSKA) Paediatric Monitoring Initiative (PAMI). The BABY‐Knee Algorithm was retrospectively applied to all patients, and corresponding scores were calculated. The predictive performance of the BABY‐Knee Algorithm was assessed using the validation approach of the original validation study and an additional failure criterion based on hard clinical endpoints. Results were compared with the previously reported results.

**Results:**

Of the 109 patients included, 78 (71.6%) were treated non‐operatively, while the remaining 31 (28.4%) underwent operative treatment. However, according to the algorithm, 34 patients (31.2%) had a score of <3, indicating non‐operative treatment, whereas 75 patients (68.8%) had a score of ≥3, indicating operative treatment. A statistically significant difference was observed between the algorithm's prediction and the performed treatment (*p* = 0.003). The original internal validation study reported a positive predictive value (PPV) of 91.7% and a negative predictive value (NPV) of 87.5%. In contrast, application of the BABY‐Knee Algorithm to the present study population resulted in a PPV of 51.1% or 31.9% and an NPV of 64.5% or 67.7%, depending on the applied definition of failure.

**Conclusion:**

The external validation of the BABY‐Knee Algorithm showed inferior predictive performance compared with the original internal validation study in an Italian population. These findings suggest that the BABY‐Knee Algorithm is not directly applicable to other populations of skeletally immature patients with ACL injuries.

**Level of Evidence:**

Level III.

## Graphical abstract

The external validation of the BABY‐Knee Algorithm showed inferior predictive performance compared with the original internal validation study in an Italian population, suggesting that the BABY‐Knee Algorithm is not directly applicable to other populations of skeletally immature patients with anterior cruciate ligament injuries.

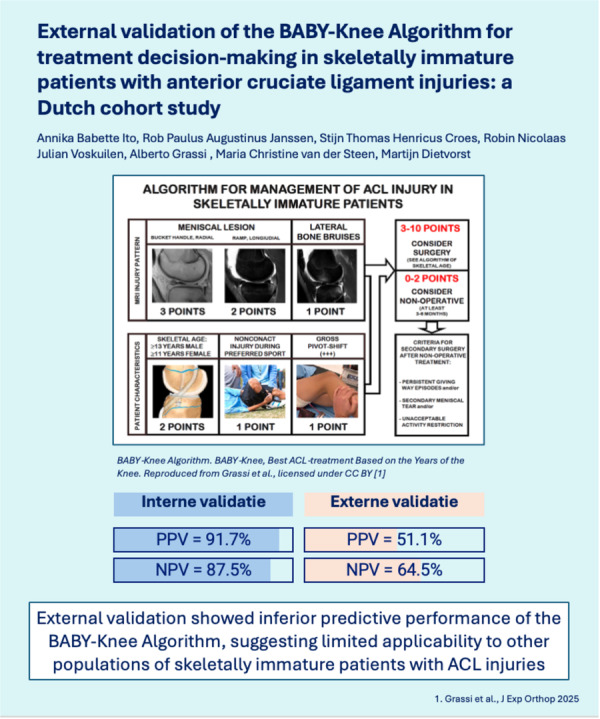

**Abbreviations**
ACL: anterior cruciate ligament
BABY‐Knee: Best ACL‐treatment Based on the Years of the Knee
BMI: body mass index
CI: confidence interval
IOC: International Olympic Committee
MRI: magnetic resonance imaging
NPV: negative predictive value
PAMI: Paediatric Anterior Cruciate Ligament Initiative
PPV: positive predictive value

## INTRODUCTION

Over the past few decades, the incidence of paediatric anterior cruciate ligament (ACL) injuries has been rising [22]. This trend has been attributed to a continuous increase in organized, competitive sports participation among children and adolescents, as well as a growing awareness [6, 20]. In these young individuals, ACL injuries can reduce quality of life [17] and increase the risk of further knee injuries and early‐onset osteoarthritis [3, 28]. The presence of open growth plates poses a unique challenge for clinicians treating paediatric ACL injuries. Historically, conservative management has been favoured due to the risk of growth disturbance and a higher incidence of graft failure following paediatric ACL reconstruction [1, 5, 20]. According to the 2018 International Olympic Committee (IOC) consensus statement, non‐operative management is recommended unless the child has repairable associated injuries requiring surgery, experiences recurrent symptomatic knee giving way after completing high‐quality rehabilitation or faces unacceptable participation restrictions. In these cases, primary ACL reconstruction is indicated.

Recent literature suggests that delaying ACL reconstruction until physeal closure increases the risk of persistent knee instability and secondary meniscal and/or cartilage injuries [1, 19, 20, 21]. Furthermore, persistent knee instability may increase the risk of sports dropout and reduced activity levels [4, 15]. This evidence has led to growing support for primary operative management. Pending consensus, an ongoing debate remains regarding the optimal treatment approach for skeletally immature patients with ACL injuries. Further research into patient‐ and injury‐related characteristics is necessary to help guide decision‐making.

In addition to the 2018 IOC consensus statement, which provides guidance for clinical decision‐making, Grassi et al. recently developed and validated the ‘BABY‐Knee Algorithm’ to support clinicians in determining the appropriate treatment approach for ACL injuries in skeletally immature patients [14]. The algorithm combines clinical and radiological variables to guide treatment decision‐making. Their internal validation showed promising results within the original retrospective Italian patient cohort. However, treatment preferences differ internationally. While early ACL reconstruction is favoured in Italy [14, 15], northern European countries tend to prefer primary non‐operative management [11, 22]. As the BABY‐Knee Algorithm was developed as a clinical decision‐support tool based on an Italian cohort, external validation in different healthcare settings is essential. Therefore, this study aimed to investigate the external validity of the BABY‐Knee Algorithm and to assess its applicability to a Dutch patient population treated in accordance with the 2018 IOC consensus statement [3].

The hypothesis was that the external validity of the BABY‐Knee Algorithm would be lower than its internal validity, suggesting the algorithm may not be fully applicable to a Dutch patient population treated in accordance with the 2018 IOC consensus statement [3].

## METHODS

### Patients

This study presents a retrospective analysis of prospectively collected data. The patients included in this study comprise the Dutch contribution to the European Society of Sport Traumatology, Knee Surgery and Arthroscopy (ESSKA) Paediatric Monitoring Initiative (PAMI) [24]. Children were included in the initiative if they had an ACL rupture confirmed by magnetic resonance imaging (MRI) and a positive Lachman's test, along with open growth plates and a skeletal age of 8–14 for females and 8–16 for males, based on an X‐ray of the left hand and evaluation according to Greulich and Pyle [16]. Patients with tibial spine fractures, combined ACL‐posterior cruciate ligament (PCL) injuries or knee dislocations were not eligible for inclusion in PAMI [24].

The present study included patients diagnosed with an ACL rupture between 1 October 2018 and 1 March 2026, who had at least 3 months of follow‐up after their initial outpatient clinic visit. Approval for the PAMI registry was obtained from the Institutional Review Board (N18.089). Written informed consent was obtained from patients and/or their legal guardians.

### Treatment

Patients were treated at Máxima Medical Centre by two orthopaedic surgeons (R. P. A. J. and M. D.), specialized in the treatment of paediatric ACL injuries. The decision between non‐operative management and primary ACL reconstruction was made after clinical and MRI evaluations, and after shared decision‐making with the patient and parents, in accordance with the 2018 IOC statement [3]. Non‐operative treatment consisted of a structured rehabilitation programme in accordance with the practice guidelines based on the International Delphi Consensus of van Melick et al. [30]. Physiotherapy was combined with a stabilizing knee brace with sport‐specific rehabilitation for at least 3 months, after which patients were re‐evaluated. If they demonstrated adequate coping without a subjective sense of instability, they were permitted to return to their chosen sports while wearing a knee brace. From that point on, the brace was mandatory for all sports and other physical activities.

Operative treatment was indicated for patients with repairable concomitant injuries requiring surgery or with unacceptable participation restrictions, in accordance with the 2018 IOC guidelines [3, 11]. The majority of patients were treated with a hybrid ACL reconstruction technique that combined a femoral all‐epiphyseal and a tibial transphyseal approach using a hamstring autograft. Any concomitant meniscus injuries present at the time of surgery were addressed during the same surgical procedure [3]. Postoperative care consisted of a standardized, physiotherapy‐based postoperative rehabilitation, also based on the International Delphi Consensus guidelines by van Melick et al. [30].

### Data collection

**Figure 1:**
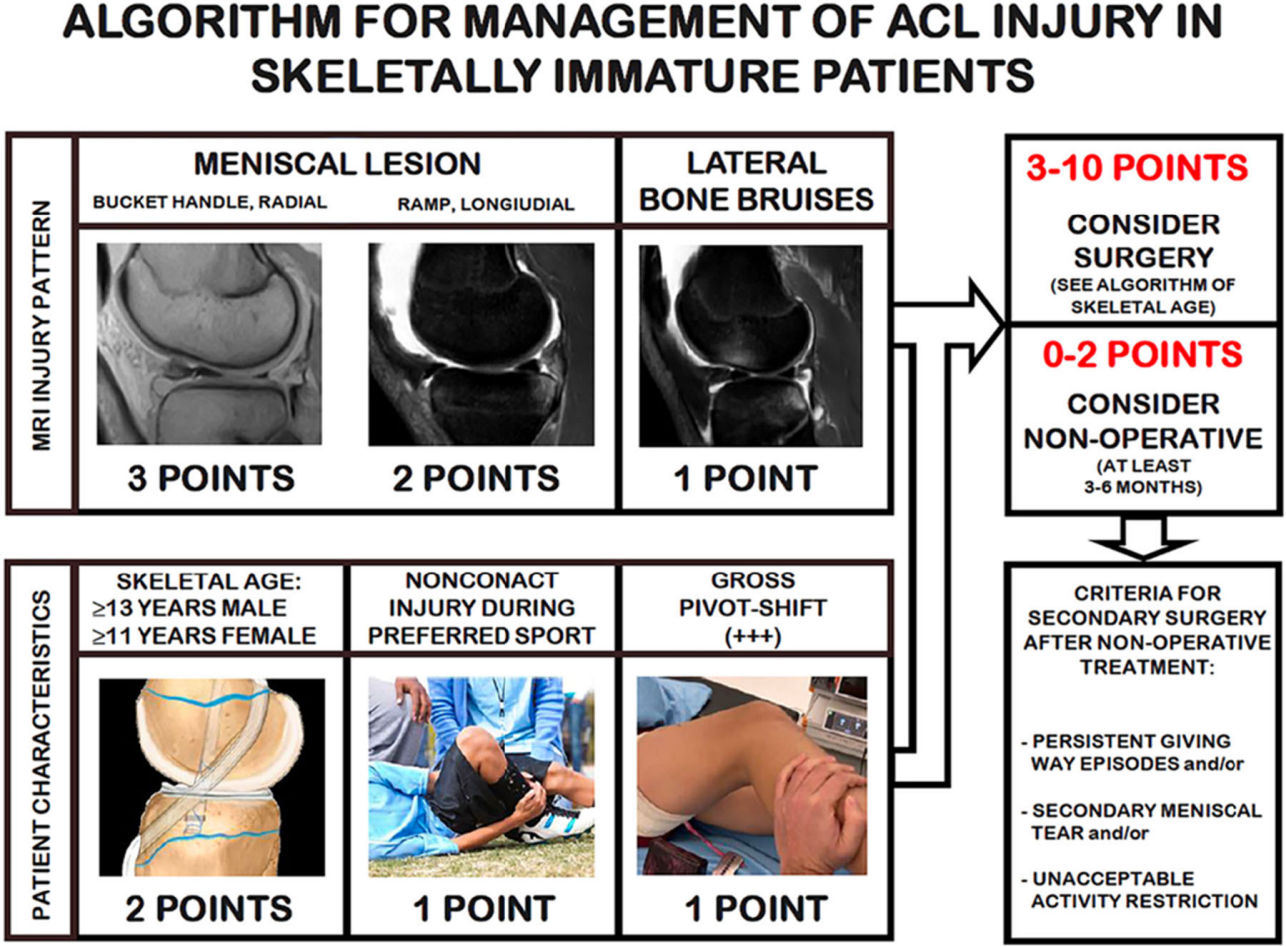
BABY‐Knee Algorithm. ACL, anterior cruciate ligament; BABY‐Knee, Best ACL‐treatment Based on the Years of the Knee. *Source*: Reproduced from Grassi et al., licensed under CC BY [14].

Data were retrospectively collected for all variables defined in the BABY‐Knee Algorithm (Figure 1) [14], as well as for variables describing the patient population, treatment and outcome. Data were retrieved from the PAMI database for all available parameters of interest [24]. Missing parameters were retrospectively collected from the electronic medical records of the included patients, and MRI scans were reviewed.

Meniscus injury was evaluated on each patient's diagnostic MRI scan by an orthopaedic surgeon with expertise in ACL reconstruction and meniscus repair (M. D.). Additionally, the presence of a bone bruise on the lateral femoral condyle or lateral tibial plateau was assessed by a trained orthopaedic resident (A. B. I.) according to the methods outlined by Ward et al. [31]. Skeletal age was determined by the same orthopaedic resident (A. B. I.) according to the shorthand MRI Bone Age Assessment Tool of the knee by Politzer et al. [26]. Data on trauma mechanisms and the grade of pivot shift were extracted from the electronic medical records. The grade of pivot shift was assessed during the initial consultation at the outpatient clinic by two experienced orthopaedic surgeons (R. P. A. J. and M. D.) and graded according to the IKDC‐2000 rating system [18]. Among patients treated primarily non‐operatively, episodes of giving‐way, inability to return to the desired level of activity without instability or development of secondary meniscus injury during the follow‐up were identified from the electronic medical records. Secondary meniscus injuries were assessed by MRI, which was performed on clinical indication.

### BABY‐Knee Algorithm

After data collection, the BABY‐Knee Algorithm was applied to all patients [14]. The algorithm consisted of three MRI‐based criteria and three patient‐characteristic criteria (Figure 1). The final algorithm score for each patient was determined by summing up the points for the identified criteria. The results were classified into two categories: 0–2 points indicating non‐operative treatment and 3–10 points indicating operative treatment.

### Analyses

#### Algorithm performance

The performance of the BABY‐Knee Algorithm was assessed according to the validation approach described by Grassi et al. [14]. Specifically, positive predictive value (PPV), negative predictive value (NPV), sensitivity and specificity were calculated for the non‐operative group. To ensure comparability with the original validation study, treatment failure and follow‐up were defined using the same criteria described by Grassi et al. [14]. Two or more giving‐way episodes, inability to return to the desired level of activity without instability or evidence of a secondary meniscus tear on a follow‐up MRI performed at least 3 months post‐injury were considered as treatment failure [14]. This definition is referred to as the ‘original failure definition’. PPV and NPV were compared with those reported by Grassi et al. in their original validation study, respectively, 91.7% and 87.5% [14]. Additionally, the performance of the BABY‐Knee Algorithm was reassessed using an alternative definition of treatment failure, referred to as the ‘alternative failure definition’. Treatment failure was based on hard clinical endpoints and defined as a secondary meniscus tear on a follow‐up MRI performed at least 3 months post‐injury and/or conversion to operative treatment before physeal closure.

#### Statistical analysis

Sensitivity, specificity, PPV and NPV of the BABY‐Knee Algorithm were calculated with 95% confidence intervals (CIs) using the Clopper–Pearson method [9]. Mann–Whitney tests, *χ* ^2^ tests or Fisher's Exact tests were used to compare algorithm components between treatment groups and between failures and successes within the non‐operative group. Means and ranges were reported for variables with a normal distribution. Medians and ranges were reported for variables that did not follow a normal distribution. The normality of continuous variables was evaluated with the Shapiro–Wilk test. Statistical analyses were performed using IBM SPSS Statistics for Windows (Version 29.0). For all analyses, statistical significance was defined as *p* < 0.05.

## RESULTS

A total of 109 skeletally immature patients with ACL injuries treated at the Máxima Medical Centre between 1 October 2018 and 1 March 2026 were included in this study. During the whole inclusion period, patients were treated in accordance with the 2018 IOC consensus statement [3]. The cohort consisted of 89 males (81.7%) and 20 females (18.3%), with a mean age of 13 years (range: 8.0–17.0). Baseline characteristics are presented in Table 1. In accordance with the 2018 IOC statement [3], 78 patients (71.6%) were treated primarily non‐operatively, while the remaining 31 (28.4%) underwent primary ACL reconstruction.

**Table 1:** Baseline characteristics of the study population.

	All patients (*N* = 109) #*N* (%) or median [min–max]	
Sex		
Male	89 (81.7)	
Female	20 (18.3)	
Age (years)	13 [8.0–17.0]	
Skeletal age		
9 years	2 (1.8)	
10 years	0 (0.0)	
10.5 years	2 (1.8)	
11 years	11 (10.1)	
11.5 years	6 (5.5)	
12 years	9 (8.3)	
13 years	21 (19.3)	
14 years	20 (18.3)	
15 years	26 (23.9)	
16 years	9 (8.3)	
17 years	0 (0.0)	
<11 years^a^	23 (2.8)	
BMI^b^	18.7 [14.8–31.4]	
Affected side		
Left	55 (50.5)	
Right	54 (49.5)	
Trauma mechanism^c^		
Non‐contact	75 (68.2)	
Contact	30 (27.3)	
Time between trauma and first outpatient clinic visit (months)	3.0 [0.0–72.0]	
Treatment		
Non‐operative	78 (71.6)	
Operative	31 (28.4)	
Follow‐up (months)	35 [4.0–88.0]	

Abbreviations: BMI, body mass index; MRI, magnetic resonance imaging.

^a^ According to the shorthand MRI Bone Age Assessment Tool of the knee by Politzer et al., the minimum category for boys is 11 years [26].

^b^ Data were missing for six patients.

^c^ Data were missing for four patients.

### BABY‐Knee Algorithm

Application of the BABY‐Knee Algorithm to the Dutch PAMI data led to the following results. According to the algorithm, 34 patients (31.2%) had a score < 3, indicating non‐operative treatment, whereas 75 patients (68.8%) had a score ≥ 3, indicating operative treatment. A statistically significant difference was observed between the algorithm's prediction and the performed treatment (*p *= 0.003). The distribution of the indicated treatment according to the BABY‐Knee Algorithm and the actual treatment received are shown in Table 2.

**Table 2:** Distribution of received treatment (operative vs. non‐operative) and suggested treatment according to BABY‐Knee classification.

	Treatment				
BABY‐KNEE	**Operative** #*N* (%)		**Non‐operative** #*N* (%)		**Total**
Non‐operative BABY‐Knee score < 3	3 (8.8)		31 (91.2)		34
Operative BABY‐Knee score ≥ 3	28 (37.3)		47 (62.7)		75
Total	31		78		

Abbreviation: BABY‐Knee, Best ACL‐treatment Based on the Years of the Knee.

Regarding the algorithm's predictive value, treatment failure was assessed in the 78 patients treated non‐operatively using the two definitions for treatment failure (Table 3). When applying the original failure definition, 21 patients met the criterion of ≥2 episodes of giving way, 5 met the criterion of inability to return to the desired level of activity without instability, and 18 developed a secondary meniscus injury. Some patients fulfilled more than one criterion for treatment failure. Among the 47 patients with a score ≥3, 24 were considered failures, resulting in a PPV of 51.1% (95% CI: 36.1–65.9). Among patients with a score <3, 11 were classified as failures, whereas the remaining 20 were successfully managed non‐operatively. Thus, resulting in an NPV of 64.5% (95% CI: 45.4–80.8). Additionally, the BABY‐Knee Algorithm demonstrated a sensitivity of 68.8% (95% CI: 50.7–83.1) and a specificity of 46.5% (95% CI: 3.12–6.23).

**Table 3:** Distribution of treatment outcomes in the non‐operatively treated patients (*n* = 78) according to BABY‐Knee classification.

	Original failure definition (Grassi et al.)					Alternative failure definition (current study)				
	Non‐operative outcome (*N* = 78)					Non‐operative outcome (*N* = 78)				
	**Failures #*N* (%)**		**Successes #*N* (%)**		**Total**	**Failures #*N* (%)**		**Successes #*N* (%)**		**Total**
BABY‐Knee score ≥ 3	24 (51.1)		23 (49.0)		47	15 (31.9)		32 (68.1)		47
BABY‐Knee score < 3	11 (35.5)		20 (64.5)		31	10 (32.3)		21 (67.7)		31
Total	35		43			25		53		

Abbreviations: ACL, anterior cruciate ligament; BABY‐Knee, Best ACL‐treatment Based on the Years of the Knee.

When applying the alternative failure definition, only 15 of the 47 patients with a BABY‐Knee score ≥3 met criteria for treatment failure, resulting in a PPV of 31.9% (95% CI: 19.1–47.1). Among patients with a BABY‐Knee score <3, 21 were successfully managed non‐operatively, resulting in an NPV of 67.7% (95% CI: 48.6–83.3). The sensitivity and specificity of the algorithm were 60.0% (95% CI: 38.7–78.9) and 39.6% (95% CI: 26.5–54.0), respectively.

The distribution of failures and successes according to the BABY‐Knee failure criteria is presented in Table 3.

### Analysis of algorithm components

In an attempt to understand the difference in performance of the BABY‐knee Algorithm within the original and current populations, the BABY‐knee Algorithm parameters were compared between patients treated non‐operatively and operatively (Table 4) and those classified as failures and successes within the non‐operative group (Table 5).

**Table 4:** Distribution of BABY‐Knee Algorithm parameters in all patients presented with respect to the received treatment.

	Operative (*N* = 31) #*N* (%) or median [min–max]		Non‐operative (*N* = 78) #*N* (%) or median [min–max]		*p* Value
*Meniscus lesion*					
Bucket handle/radial	6 (19.4)		0 (0.0)		<0.001*
Ramp/longitudinal	22 (71.0)		12 (15.4)		<0.001*
Lateral bone bruise	17 (54.8)		36 (46.2)		0.41
Skeletal age ≥ 13 males or ≥ 11 females	24 (77.4)		58 (74.4)		0.74
Non‐contact injury during preferred sport	7 (22.6)		39 (50.0)		0.01*
Gross pivot shift	2 (6.5)		1 (1.3)		0.19
‘BABY‐Knee’ score	5 [2.0–8.0]		3 [0.0–6.0]		<0.001*

Abbreviations: ACL, anterior cruciate ligament; BABY‐Knee, Best ACL‐treatment Based on the Years of the Knee.

* Statistically significant difference (*p* < 0.05).

**Table 5:** Distribution of BABY‐Knee Algorithm parameters in non‐operatively treated patients (*n* = 78) presented with respect to outcome, failure versus treatment success.

	Original failure definition (Grassi et al.)					Alternative failure definition (current study)				
	Failures (*N* = 35) #*N* (%) or median [min–max]		Success (*N* = 43) #*N* (%) or median [min–max]		*p* Value	Failures (*N* = 35) #*N* (%) or median [min–max]		Success (*N* = 43) #*N* (%) or median [min–max]		*p* Value
*Meniscus lesion*										
Bucket handle/radial	0 (0.0)		0 (0.0)		1.00	0 (0.0)		0 (0.0)		1.00
Ramp/longitudinal	9 (25.7)		3 (7.0)		0.02*	7 (28)		6 (11.3)		0.10
Lateral bone bruise	15 (42.9)		21 (48.8)		0.60	8 (32.0)		28 (52.8)		0.09
Skeletal age ≥ 13 males or ≥ 11 females	27 (77.1)		31 (72.1)		0.61	19 (76.0)		39 (73.6)		0.82
Non‐contact injury during preferred sport	19 (54.3)		20 (46.5)		0.50	13 (52)		26 (49.1)		0.80
Gross pivot shift	1 (2.9)		0 (0.0)		0.50	1 (4.0)		0 (0.0)		0.32
‘BABY‐Knee’ score	3 [0.0–5.0]		3 [0.0–6.0]		0.10	3 [0.0–5.0]		3 [0.0–6.0]		0.43

Abbreviation: ACL, anterior cruciate ligament; BABY‐Knee, Best ACL‐treatment Based on the Years of the Knee.

* Statistically significant difference (*p* < 0.05).

As expected, given the indication for operative treatment according to the IOC statement [3], the incidence of bucket‐handle and radial meniscus lesions, as well as ramp and longitudinal tears, was higher in patients treated operatively than in those treated non‐operatively (*p* < 0.001 and *p* < 0.001, respectively).

Notably, despite having concomitant ramp or longitudinal meniscus tears at their initial diagnostic MRI, 12 patients (15.4%) received primary conservative treatment. Four patients had a meniscus injury on their diagnostic MRI, which had healed on an additional MRI performed at the first outpatient clinical visit at the Máxima Medical Centre. The remaining 8 patients had degenerative or very small stable tears on MRI that could be managed non‐operatively and therefore did not serve as an indication for primary ACL reconstruction.

Furthermore, patients in the non‐operative group had a significantly higher incidence of non‐contact injury during preferred sports (*p* = 0.01).

When the original failure definition was applied, the only significant difference between failures and successes in the non‐operative group was a higher incidence of concomitant ramp or longitudinal meniscus injuries on the diagnostic MRI among failures (*p* = 0.02). However, these lesions were not responsible for the failure classification during follow‐up. When the alternative failure definition was applied, no statistically significant differences in algorithm parameters were observed between failures and successes.

## DISCUSSION

The most important finding of the present study is that the external validation of the BABY‐Knee Algorithm in a Dutch cohort of skeletally immature patients with ACL injuries showed reduced predictive ability compared with that reported in the original internal validation study [14]. This suggests that its value as a decision‐support tool is lower in this study's cohort compared with the original Italian population.

The original validation study reported a PPV of 91.7%, indicating that nearly all patients for whom the algorithm recommended operative treatment but who received non‐operative treatment failed this approach. In contrast, when the algorithm was applied to the patient cohort of the present study, only half of the patients for whom the algorithm recommended operative treatment failed non‐operative treatment (PPV: 51.1%) when the original failure definition was used. Regarding the NPV, the difference between external and internal validation was smaller than that observed for the PPV. However, external validation still showed a lower NPV (64.5%) than the internal validation (87.5%). In addition, both sensitivity and specificity were also lower in the external validation cohort (68.6% and 64.5%, respectively) compared with the internal validation results (91.7% and 87.5%, respectively). The algorithm performed even less in the current patient cohort when the alternative failure definition was applied. Thus, the algorithm's accuracy in predicting which patients would fail conservative treatment and which would not require primary ACL reconstruction was lower, according to the external validation, than that reported in the original internal validation study. This indicates variability in the algorithm's performance across patient populations.

This difference in predictive value may be attributed to the variation in treatment approaches. In the study by Grassi et al., only 26.7% of patients received non‐operative treatment [14], compared to 71.6% in the present study's cohort. In the present study, non‐operative treatment was used more often and supported by a structured rehabilitation programme, in line with the practice guidelines based on the International Delphi Consensus of van Melick et al. [30], combined with a stabilizing knee brace. This more standardized non‐operative management approach may have contributed to the differences observed between studies. Furthermore, since a smaller proportion of patients was treated non‐operatively in the original validation study, the PPV and NPV calculations were based on a smaller number of patients, potentially limiting the reliability of the reported estimates. Another important factor to consider is variation in patient population, including the type of sports and the willingness to return to the pre‐injury level of participation. This aspect is difficult to standardize without carefully assessing patients' and families' expectations, level of play, and the psychological drawbacks associated with sport cessation or performance decline, especially in organized team sports like soccer. Without assessing the ability to cope with instability by decreasing the level of sport to a non‐pivoting activity, or by enhancing neuromuscular control, it is difficult to determine the exact failure or success of conservative treatment, and thus the generalizability across populations from different environments.

The predictive accuracy of the BABY‐Knee Algorithm was calculated based on the proportion of treatment failures and successes among patients treated non‐operatively [14]. According to Grassi et al., treatment failure was defined as two or more giving‐way episodes, inability to return to the desired level of activity without instability or evidence of a secondary meniscus tear on a follow‐up MRI performed at least 3 months post‐injury. Within the original validation study, 60.0% of patients treated nonoperatively were considered treatment failures (12/20) under these failure criteria, compared to 44.9% in the present study's cohort (35/78). However, the original failure definition may have resulted in a higher number of patients being classified as treatment failures. While secondary meniscus injury is a well‐established failure criterion for non‐operative treatment of paediatric ACL injuries [1, 19, 20], giving way episodes and returning to the desired level of activity without instability leave room for interpretation. According to the IOC statement, ACL reconstruction following non‐operative treatment is recommended in case of repairable associated injuries requiring surgery, recurrent symptomatic knee giving way after high‐quality rehabilitation or unacceptable participation restrictions [3]. While Grassi et al. followed these indications and aimed to further standardize and specify them to enable an objective classification of failure [14], this approach may have resulted in an overestimation of the treatment failure rate. Some patients might still cope with non‐operative treatment despite meeting the original failure criteria by Grassi et al. For instance, patients may still successfully undergo non‐operative treatment despite more than two giving‐way episodes and might accept certain adaptations or concessions in sports participation. Therefore, in this study, an alternative definition of failure was also applied, using ‘conversion to operative treatment before physeal closure’ and/or ‘secondary meniscus injury’. With this alternative definition of failure, only 32.1% (25/78) of patients were considered treatment failures. This resulted in a PPV of 31.9%, an NPV of 67.7%, a sensitivity of 60.0% and a specificity of 39.6%. As a lesser proportion of the patients who, according to the BABY‐Knee algorithm, had an operation indication (≥3) failed, the predictive performance of the BABY‐Knee Algorithm was not enhanced. However, direct comparison is not possible, as the exact number of treatment failures according to the alternative definition of failure in the original Italian cohort is unknown.

The BABY‐Knee Algorithm places significant emphasis on the presence of concomitant meniscus injuries as an indicator for operative treatment. Untreated meniscus injuries in children with ACL injuries increase the risk of knee instability and early‐onset osteoarthritis [3, 27]. Consistent with the algorithm and the 2018 IOC statement [3], the presence of repairable concomitant meniscus injuries requiring surgery served as a primary indicator for operative treatment within the cohort of the present study. Accordingly, patients who underwent operative treatment had a significantly higher incidence of meniscus injuries compared to those treated nonoperatively.

Bucket‐handle and radial meniscus tears are absolute indicators of operative treatment according to the BABY‐Knee Algorithm, each counting for 3 points, placing a patient immediately above the operative treatment threshold [14]. Such tears considerably affect knee biomechanics, leading to loss of meniscal load transmission and increased risk of overload‐induced cartilage damage [7, 12, 32]. Because of the increased risk of future degenerative joint changes, all patients in the present study cohort presenting with concomitant bucket‐handle or radial tears also received operative treatment.

Longitudinal meniscus injuries are more common among children and adolescents [2]. Compared to bucket‐handle and radial meniscus tears, longitudinal and ramp lesions have a lower biomechanical impact. This leaves room for non‐operative treatment of small stable non‐displaced concomitant meniscus tears in selected cases. Additionally, the rich vascularization of the meniscus in children contributes to the great healing potential of peripheral tears [29]. In the present study, 30.0% of patients who presented with a meniscus injury at baseline demonstrated complete healing on follow‐up MRI or at arthroscopy. Both the internal validation study by Grassi et al. and the present study found a significantly higher incidence of ramp or longitudinal meniscus lesions among patients who failed non‐operative treatment than among those who were successful. This finding may question the room for non‐operative treatment in patients with concomitant meniscus injuries. Nevertheless, in the present study, none of these meniscus lesions were responsible for the failure classification during follow‐up. Among the nine patients classified as failures with a ramp or longitudinal meniscus injury at baseline, two failed due to >2 giving‐way episodes, whereas seven failed due to a secondary meniscus injury. Notably, all represented new meniscus injuries rather than progression of the initial lesion.

Higher skeletal age was another strong contributor to the decision for operative treatment in the BABY‐Knee Algorithm [14]. Previous literature has also reported that skeletal age influences treatment decisions for skeletally immature patients with ACL injuries [5, 8, 15]. Higher skeletal age indicates less remaining growth, which is associated with a reduced risk of growth disturbances, one of the main complications of paediatric ACL reconstruction [13, 23]. Therefore, primary ACL reconstruction becomes more attractive as skeletal age increases. Consistent with the algorithm's rationale, the incidence of patients with a skeletal age of ≥12 in males and ≥11 in females was significantly higher among those treated operatively in the original validation study. Furthermore, a significantly higher rate of non‐operative treatment failure among patients with a skeletal age ≥12 in males and ≥11 in females was reported, supporting the inclusion of this criterion within the algorithm. In contrast to Grassi et al., the present study found no significant difference in treatment performed or in the outcome of non‐operative treatment based on skeletal age (≥13 for males or ≥11 for females). This suggests that the weight applied to skeletal age as a criterion within the algorithm may be overstated. While physeal status remains a key consideration in skeletally immature patients and is closely linked to skeletal age, our findings suggest that skeletal age should contribute to the overall treatment decision‐making process rather than serve as a determining factor.

This study had several limitations. Despite data being prospectively collected in the PAMI registry, additional data extraction from medical records was necessary because the registry currently lacks data for all variables in the BABY‐Knee Algorithm. This process might have introduced information bias. Second, treatment decisions were based on the 2018 IOC consensus statement [3], which partially overlaps with the BABY‐Knee Algorithm criteria. This overlap may have influenced the observed performance, thereby potentially introducing incorporation bias. Additionally, complete data on return to pre‐injury sport participation were not available for all included patients. Therefore, comparison of activity levels after treatment between cohorts was not possible. Furthermore, a proportion of patients with meniscus injuries were managed non‐operatively. Although this approach was in line with the IOC statement [3], which considers only repairable associated injuries requiring surgery as an indicator for operative treatment, it may have introduced heterogeneity in the initial treatment strategies within the cohort. Furthermore, given the importance of concomitant meniscus injury as an indicator for operative treatment, another limitation is the limited sensitivity of MRI‐based diagnosis of meniscus injury in the paediatric population [10, 25]. Consequently, some meniscus lesions may have been missed, potentially resulting in misclassification. Lastly, predictive values should be interpreted with caution, as they are prevalence‐dependent and retrospectively applied, therefore influenced by differences in treatment strategies between cohorts.

The ongoing debate regarding the optimal treatment approach for skeletally immature patients with ACL injuries emphasizes the need for a decision support system that can help guide clinical decision‐making. Although the BABY‐Knee Algorithm lacks generalizability across patient populations, the included variables appear relevant to guide decision support. In the present study, operatively treated patients had higher BABY‐Knee Algorithm scores than patients treated non‐operatively. However, with the current threshold for operative treatment, the algorithm's predictive value was lower when applied to a different patient population. Lack of generalizability may be due to differences in treatment approaches, healthcare systems, reimbursement structures and cultural factors. Additionally, emphasis on sport participation, availability and acceptance of brace use during organized sports, and patients' willingness to adapt or accept concessions within sport participation may vary between populations. Future adaptations of the algorithm might therefore profit from a better understanding of differences in treatment and decision‐making processes across centres. Furthermore, future use of multi‐centre cohorts can ensure applicability across different clinical settings, improving decision support tools. The full PAMI registry, launched by the ESSKA, provides such a database [24].

## CONCLUSION

The external validation of the BABY‐Knee Algorithm shows inferior predictive performance compared with the original internal validation study in an Italian population [14]. These findings suggest that the BABY‐Knee Algorithm is not directly applicable to other populations of skeletally immature patients with ACL injuries. Differences in sport participation, cultural diversity, treatment indications and management strategies between centres/countries may explain the variability in the algorithm's performance across patient populations and should be further investigated to facilitate the development of more generalizable decision support tools.

## AUTHOR CONTRIBUTIONS

Annika Babette Ito, Rob Paulus Augustinus Janssen, Robin Nicolaas Julian Voskuilen, Maria Christine van der Steen and Martijn Dietvorst contributed to the conception and design of the study. Annika Babette Ito, Stijn Thomas Henricus Croes and Robin Nicolaas Julian Voskuilen participated in data acquisition. Annika Babette Ito and Stijn Thomas Henricus Croes analysed the data. Annika Babette Ito drafted the manuscript. Annika Babette Ito, Rob Paulus Augustinus Janssen, Maria Christine van der Steen and Martijn Dietvorst were involved in data interpretation. Annika Babette Ito, Rob Paulus Augustinus Janssen, Alberto Grassi, Maria Christine van der Steen and Martijn Dietvorst critically revised the manuscript. Rob Paulus Augustinus Janssen, Maria Christine van der Steen and Martijn Dietvorst supervised the research project. All authors have read and approved the final version of the manuscript and agree to be responsible for all aspects of the work, ensuring that any questions related to the accuracy or integrity of any part are properly investigated and resolved.

## FUNDING INFORMATION

The authors have no funding to report.

## CONFLICT OF INTEREST STATEMENT

The authors declare no conflicts of interest.

## ETHICS STATEMENT

The Medical Ethical Committee at the Máxima Medical Centre determined that the rules laid down in the Medical Research Involving Human Subjects Act (MWO) do not apply to this study. The local ethics committee of the Máxima Medical Centre approved this study (Reference number: N.18.089). Informed consent was obtained from all patients and/or their legal guardians prior to treatment for the scientific use of clinical data as part of standard care.

## Data Availability

The data supporting the findings of this study are available upon request from the corresponding author (Annika Babette Ito). The data are not publicly available due to privacy or ethical restrictions.

## References

[1] Acquaah F , Fontalis A , Collier W , Lisacek‐Kiosoglous AB , Ahluwalia A , Dalrymple J , et al. Current approaches to diagnosing and managing anterior cruciate ligament injuries in skeletally immature patients. Bone Joint J. 2026;108‐B(1):9–20. 10.1302/0301-620X.108B1.BJJ-2024-1559.R2 41475360

[2] Araújo FF , Guimarães JB , da Cruz IAN , Morimoto LR , Filho AGO , Nico MAC . Pediatric menisci: normal aspects, anatomical variants, lesions, tears, and postsurgical findings. Insights Imaging. 2024;15(1):295. 39666127 10.1186/s13244-024-01867-6 PMC11638453

[3] Ardern CL , Ekås G , Grindem H , Moksnes H , Anderson A , Chotel F , et al. 2018 International Olympic Committee consensus statement on prevention, diagnosis and management of paediatric anterior cruciate ligament (ACL) injuries. Knee Surg Sports Traumatol Arthrosc. 2018;26(4):989–1010. 29455243 10.1007/s00167-018-4865-y PMC5876259

[4] Attri M , D'Ambrosi R , Farinelli L , Malik SS , De Sa D , Tapasvi S , et al. ACL reconstruction in skeletally immature athletes: current concepts. Medicina. 2025;61(4):562. 40282853 10.3390/medicina61040562 PMC12028375

[5] Bixby EC , Heyworth BE . Management of anterior cruciate ligament tears in skeletally immature patients. Curr Rev Musculoskelet Med. 2024;17(7):258–272. 38639870 10.1007/s12178-024-09897-9 PMC11156825

[6] Bram JT , Magee LC , Mehta NN , Patel NM , Ganley TJ . Anterior Cruciate ligament injury incidence in adolescent athletes: a systematic review and meta‐analysis. Am J Sports Med. 2021;49(7):1962–1972. 33090889 10.1177/0363546520959619

[7] Canton G , Maritan G , Impellizzeri F , Formentin C , Murena L . Bucket‐handle meniscal tears in children under the age of 10: a literature review. Acta Biomed. 2021;92(S3):e2021020. 10.23750/abm.v92iS3.11742 PMC8420820 34313663

[8] Christino MA , Hutchinson LE , Pennock AT , Cook DL , Anderson CN , Busch MT , et al. Descriptive epidemiology of complete ACL tears in the skeletally immature population: a prospective multicenter PLUTO study. Am J Sports Med. 2025;53(3):612–622. 39876031 10.1177/03635465241312215

[9] Clopper CJ , Pearson ES . The use of confidence or fiducial limits illustrated in the case of the binomial. Biometrika. 1934;26(4):404–413.

[10] Dawkins BJ , Kolin DA , Park J , Fabricant PD , Gilmore A , Seeley M , et al. Sensitivity and specificity of MRI in diagnosing concomitant meniscal injuries with pediatric and adolescent acute ACL tears. Orthop J Sports Med. 2022;10(3):23259671221079338. 35295551 10.1177/23259671221079338 PMC8918745

[11] Dietvorst M , Reijman M , van Zutven R , van den Bekerom MPJ , Meuffels DE , Somford MP , et al. Current state of care for pediatric ACL ruptures in the Netherlands: a survey. J Knee Surg. 2021;34(5):520–525. 31550739 10.1055/s-0039-1697626

[12] Feeley BT , Lau BC . Biomechanics and clinical outcomes of partial meniscectomy. J Am Acad Orthop Surg. 2018;26(24):853–863. 30247309 10.5435/JAAOS-D-17-00256

[13] Fury MS , Paschos NK , Fabricant PD , Anderson CN , Busch MT , Chambers HG , et al. Assessment of skeletal maturity and postoperative growth disturbance after anterior cruciate ligament reconstruction in skeletally immature patients: a systematic review. Am J Sports Med. 2022;50(5):1430–1441. 33984243 10.1177/03635465211008656

[14] Grassi A , Borque K , Dietvorst M , Altovino E , Rossi C , Ambrosini L , et al. Creation and validation of a treatment algorithm for skeletally immature patients with acute anterior cruciate ligament injury based on MRI and patient characteristics. J Exp Orthop. 2025;12(3):e70280. 40766807 10.1002/jeo2.70280 PMC12322690

[15] Grassi A , Borque K , Rossi C , Cascone B , Della Villa F , Zaffagnini S . Current concept on assessment and management of anterior cruciate ligament injury in skeletally immature athletes. J Exp Orthop. 2025;12(4):e70498. 41255741 10.1002/jeo2.70498 PMC12620561

[16] Greulich W , Pyle S . Radiographic atlas of skeletal development of the hand and wrist. 2nd ed. Palo Alto, CA: Stanford University Press; 1959.

[17] Hansen CF , Brodersen JB , Krogsgaard MR . Health‐related quality of life for children with anterior cruciate ligament deficiency: ensuring content validity of the new KIDS‐KNEES‐ACL questionnaire. Knee Surg Sports Traumatol Arthrosc. 2025;33(2):555–566. 39101228 10.1002/ksa.12393

[18] Hefti F , Müller W , Jakob RP , Stäubli HU . Evaluation of knee ligament injuries with the IKDC form. Knee Surg Sports Traumatol Arthrosc. 1993;1(3–4):226–234. 8536037 10.1007/BF01560215

[19] James EW , Dawkins BJ , Schachne JM , Ganley TJ , Kocher MS , Anderson CN , et al. Early operative versus delayed operative versus nonoperative treatment of pediatric and adolescent anterior cruciate ligament injuries: a systematic review and meta‐analysis. Am J Sports Med. 2021;49(14):4008–4017. 33720764 10.1177/0363546521990817

[20] Jin H , Tahir N , Jiang S , Mikhail H , Pavel V , Rahmati M , et al. Management of anterior cruciate ligament injuries in children and adolescents: a systematic review. Sports Med Open. 2025;11(1):40. 40263204 10.1186/s40798-025-00844-7 PMC12014893

[21] Kolin DA , Dawkins B , Park J , Fabricant PD , Gilmore A , Seeley M , et al. ACL reconstruction delay in pediatric and adolescent patients is associated with a progressive increased risk of medial meniscal tears. J Bone Jt Surg. 2021;103(15):1368–1373. 10.2106/JBJS.20.01459 34156989

[22] Kooy CEW , Jakobsen RB , Fenstad AM , Persson A , Visnes H , Engebretsen L , et al. Major increase in incidence of pediatric ACL reconstructions from 2005 to 2021: a study from the Norwegian Knee Ligament Register. Am J Sports Med. 2023;51(11):2891–2899. 37497771 10.1177/03635465231185742 PMC10478322

[23] Longo UG , Villa Corta M , Valente F , Ruzzini L , D'Hooghe P , Samuelsson K , et al. Skeletal maturity assessment in pediatric ACL‐reconstruction. Children. 2025;12(9):1186. 41007051 10.3390/children12091186 PMC12468475

[24] Mouton C , Moksnes H , Janssen R , Fink C , Zaffagnini S , Monllau JC , et al. Preliminary experience of an international orthopaedic registry: the ESSKA Paediatric Anterior Cruciate Ligament Initiative (PAMI) registry. J Exp Orthop. 2021;8(1):45. 34173077 10.1186/s40634-021-00366-7 PMC8233435

[25] Munger AM , Gonsalves NR , Sarkisova N , Clarke E , VandenBerg CD , Pace JL . Confirming the presence of unrecognized meniscal injuries on magnetic resonance imaging in pediatric and adolescent patients with anterior cruciate ligament tears. J Pediatr Orthop. 2019;39(9):e661–e667. 30628976 10.1097/BPO.0000000000001331

[26] Politzer CS , Bomar JD , Pehlivan HC , Gurusamy P , Edmonds EW , Pennock AT . Creation and validation of a shorthand magnetic resonance imaging bone age assessment tool of the knee as an alternative skeletal maturity assessment. Am J Sports Med. 2021;49(11):2955–2959. 34347536 10.1177/03635465211032986

[27] Pruneski JA , Tavabi N , Heyworth BE , Kocher MS , Kramer DE , Christino MA , et al. Prevalence and predictors of concomitant meniscal surgery during pediatric and adolescent ACL reconstruction: analysis of 4729 patients over 20 years at a tertiary‐care regional children's hospital. Orthop J Sports Med. 2024;12(3):23259671241236496. 38515604 10.1177/23259671241236496 PMC10956158

[28] Snoeker B , Turkiewicz A , Magnusson K , Frobell R , Yu D , Peat G , et al. Risk of knee osteoarthritis after different types of knee injuries in young adults: a population‐based cohort study. Br J Sports Med. 2020;54(12):725–730. 31826861 10.1136/bjsports-2019-100959

[29] Tian Y , Shen YL , Wang KL , Guo DW , Hou TT , Ma T . Advances in repair of non‐discoid meniscus injuries in children: a narrative review. Front Pediatr. 2025;13:1674832. 41695118 10.3389/fped.2025.1674832 PMC12894249

[30] van Melick N , Dietvorst M , van Oort MIAM , Claessens RLA , Janssen RPA , Bogie R , et al. Anterior cruciate ligament rehabilitation for the 10‐ to 18‐year‐old adolescent athlete: practice guidelines based on International Delphi Consensus. Orthop J Sports Med. 2023;11(7):23259671231172454. 37492781 10.1177/23259671231172454 PMC10363891

[31] Ward P , Chang P , Radtke L , Brophy RH . Clinical implications of bone bruise patterns accompanying anterior cruciate ligament tears. Sports Health. 2022;14(4):585–591 34231443 10.1177/19417381211029583 PMC9214899

[32] Zhang J , Chen B , Chen B , Wang H , Han Q , Tang X , et al. Clinical application of finite element analysis in meniscus diseases: a comprehensive review. Arch Comput Methods Eng. 2025;32(7):4163–4195

